# Validation of a New Handheld Automated Fixed-Force Applanation Tonometer

**DOI:** 10.1167/tvst.15.7.21

**Published:** 2026-07-14

**Authors:** Fiona S. McDonnell, Erin Simons, Michael Zhu, W. Daniel Stamer, Joanne C. Wen

**Affiliations:** 1 Department of Ophthalmology and Visual Sciences, University of Utah, Salt Lake City, UT, USA; 2 Blur Product Development, Cary, NC, USA; 3 Duke Eye Center, Duke University, Durham, NC, USA

**Keywords:** Tonometer, tonometry, intraocular pressure, applanation tonometry, automated

## Abstract

**Purpose:**

To validate a novel tonometer on ex vivo human eyes.

**Methods:**

A device was constructed to apply a known and constant force using a custom tonometer tip, and the resulting applanation image is recorded with a camera. Software automatically measures the applanation area and converts to an intraocular pressure (IOP). Ex vivo human eyes were cannulated, and the IOP manometrically was set to 5 to 45 mm Hg in 5-mm Hg increments. At each IOP, a 5- to 10-second recording was obtained with the device and converted to an IOP. Measured IOPs were compared to the set IOP value.

**Results:**

Eight ex vivo human eyes were included in this study. The mean difference (± SD) and mean absolute difference (± SD) between the stable gravity-induced manometric IOP and the measured IOP were –1.2 ± 4.5 mm Hg and 3.1 ± 3.5 mm Hg, respectively. Within the 10- to 30-mm Hg range, the mean difference and absolute difference were 0.7 ± 1.7 mm Hg and 1.4 ± 1.1 mm Hg, respectively. The prototype IOP and the set IOP showed a significant linear correlation across the full range of set IOPs (*R*^2^ = 0.90, *P* < 0.0001), which improved further for the more clinically relevant range of 10- to 30-mm Hg set IOPs (*R*^2^ = 0.95, *P* < 0.0001).

**Conclusions:**

Compared to clamped IOP, measurements using a novel handheld automated fixed-force applanation tonometer demonstrated high accuracy, especially within the 10- to 30-mm Hg range.

**Translational Relevance:**

We present a novel tonometer validated on ex vivo human eyes, which demonstrates high accuracy and may have applicability to patient care.

## Introduction

The measurement of intraocular pressure (IOP), referred to as tonometry, is an integral element of the ophthalmic exam. Elevated IOP is the greatest modifiable risk factor for the development of glaucoma, the most common cause of irreversible blindness worldwide.^1^^,^^2^ Furthermore, when glaucoma is diagnosed, nearly all treatments, whether medical, procedural, or surgical, aim to reduce IOP.^3^^,^^4^ Accurate and reliable tonometry is therefore crucial in the diagnosis and management of glaucoma.

Tonometers were developed and introduced in the late 19th century using various approaches, including applanation and indentation.^5^ All forms of applanation-based tonometry rely on the underlying assumptions of the Imbert–Fick principle, where the pressure inside an infinitely thin-walled sphere can be calculated by flattening the sphere with a known force and measuring the resulting flattened area, or vice versa.^6^^,^^7^ Early iterations of applanation-based tonometry relied on a “fixed force,” where, under gravity, a known weight was applied to the supine eye and the resulting applanation area was estimated and converted to an IOP.^5^^,^^8^ With the introduction of Goldmann applanation tonometry (GAT) in 1954 and the use of the split prism, these fixed-force methods fell out of favor and fixed-area applanation (where the force is adjusted to a set area) became the clinical gold standard.^5^ GAT has since been used in numerous large randomized multi-center trials and is considered a reference standard for new tonometer development.^9^^–^^11^

GAT attaches to the standard slit-lamp microscope used in ophthalmic exams and is limited in portability and in patient positioning. In response, the Perkins tonometer was developed in 1965 to facilitate a portable version of applanation tonometry that could also be used on patients in the supine position.^12^ Subsequent studies demonstrated strong correlations between GAT and Perkins tonometer measurements.^13^^–^^16^ However, applanation-based tonometry has a steep learning curve, with higher variability in measurements, especially in less-experienced trainees.^17^ The subjective alignment of applanation mires has also been shown to introduce systematic bias into the measurements, as evidenced by a preference for even numbers, likely due to the even tick marks on the applanation dials.^18^^,^^19^ There is also a higher incidence of symmetric IOP measurements between eyes compared to more objective tonometry methods.^20^

To address these limitations, we developed a portable and objective method of performing applanation-based tonometry. In this study, we constructed a handheld fixed-force applanation tonometer and tested the prototype on ex vivo human donor globes perfused at a range of IOPs to assess device accuracy.

## Methods

### Construction of the Handheld Fixed-Force Applanation Tonometer

**Figure 1.**
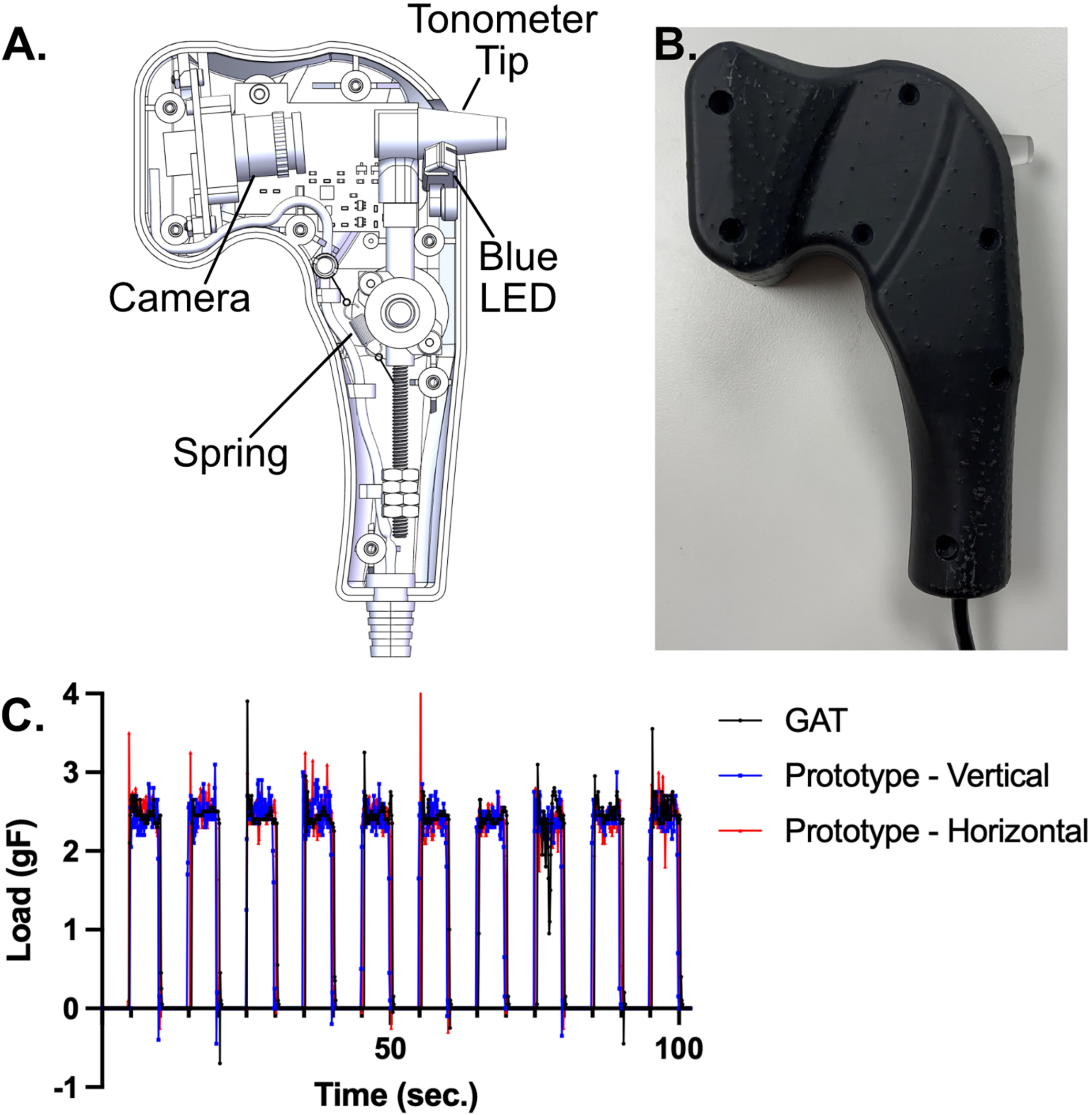
Prototype fixed-force applanation tonometer. (**A**) Prototype schematic showing a camera aligned with a tonometer tip set in an arm attached to a spring under blue LED illumination. (**B**) Assembled prototype used in this study. (**C**) Force output recordings of the prototype in both vertical and horizontal positions were confirmed to be comparable to the force output of a Goldmann applanation tonometer set to 2.5 gF (the equivalent force to measure an IOP of 25 mm Hg).

The construction of the device is shown in Figure 1A. A custom tonometer tip using medical-grade polymethyl methacrylate (PMMA) was machined to match the standard GAT tonometer tip dimensions but without the internal split-prism feature. This custom tip was polished to optical clarity and inserted into the arm of the constant-force portion of the device. The device applies a constant force using a lever–pivot mechanism. The applanation prism is connected to a lever arm. A spring and a counterweight are connected to the lever arm on the other side of the pivot point. The geometry is tuned so that, as the applanation arm rotates, the spring stretches and the spring force increases, but the lever arm of the spring (perpendicular distance from the pivot to the force line of the spring) shrinks, so the spring moment stays nearly constant. Gravity moments also shift as the applanation arm rotates, but the counterweight position is tuned to partially compensate for the residual variation in spring moment. Free body diagrams for constant force in the vertical/upright and horizontal/supine positions, as well as a force equilibrium analysis, are shown in a Supplementary Figure and Supplementary Excel file, respectively. The spring creating the constant force is made of music wire (steel, ASTM A228) with a maximum load of 85.4 gram-force (gF) (0.1882 lb) and a maximum deflection of 2.75 inches in extension before deforming. The device was tuned to create a force equivalent of 2.5 gF as this allows a theoretical lower limit of a 5-mm Hg measurable IOP given the diameter of the custom tonometer tip. A digital camera (UC50MPB_L160, Spinel Electronics, Irvine, CA) was then aligned with the tonometer tip to allow recording of the applanation surface through the tonometer tip. Blue light-emitting diode (LED) lights were installed to illuminate the tonometer tip and facilitate identification of the fluorescein-stained applanation mire. The entire prototype was housed in a custom three-dimensional (3D)-printed case (Fig. 1b). The device is connected to a laptop computer via Universal Serial Bus (USB) to view and record video. The device is powered by the same USB cord (5 V).

When the prototype had been assembled, the constant force output by the prototype was assessed with a force gauge (M3-012; Mark-10 Corporation, Copiague, NY), and the force data were recorded and exported using Mark-10 MESUR Lite software. The force gauge was rested near the edge of a flat table. To facilitate exertion of a consistent force by the device, the operator used the last fourth and fifth digits of their hand to stabilize their hand and the handheld device on the table edge while contacting the force meter. This was felt to be analogous to stabilizing the hand on the cheek of a face while obtaining a measurement. The applanation tip of the prototype contacted the tip of the force gauge to record the force output for 5 seconds. To compare, a standard GAT was loaded with a standard applanation prism, and the GAT dial was set to 2.5 gF. The GAT was positioned in front of the force gauge such that the front face of the applanation prism and the front face of the force gauge loading shaft were parallel to each other. The force gauge was moved forward such that the loading shaft contacted the applanation prism for 5 seconds. No adjustment to the GAT dial was made during contact with the force gauge. This was repeated 10 times each for the prototype device in both the vertical/upright position and the horizontal/supine position and for the GAT.

### Validation of Tonometer on Ex Vivo Human Cadaveric Eyes

**Figure 2.**
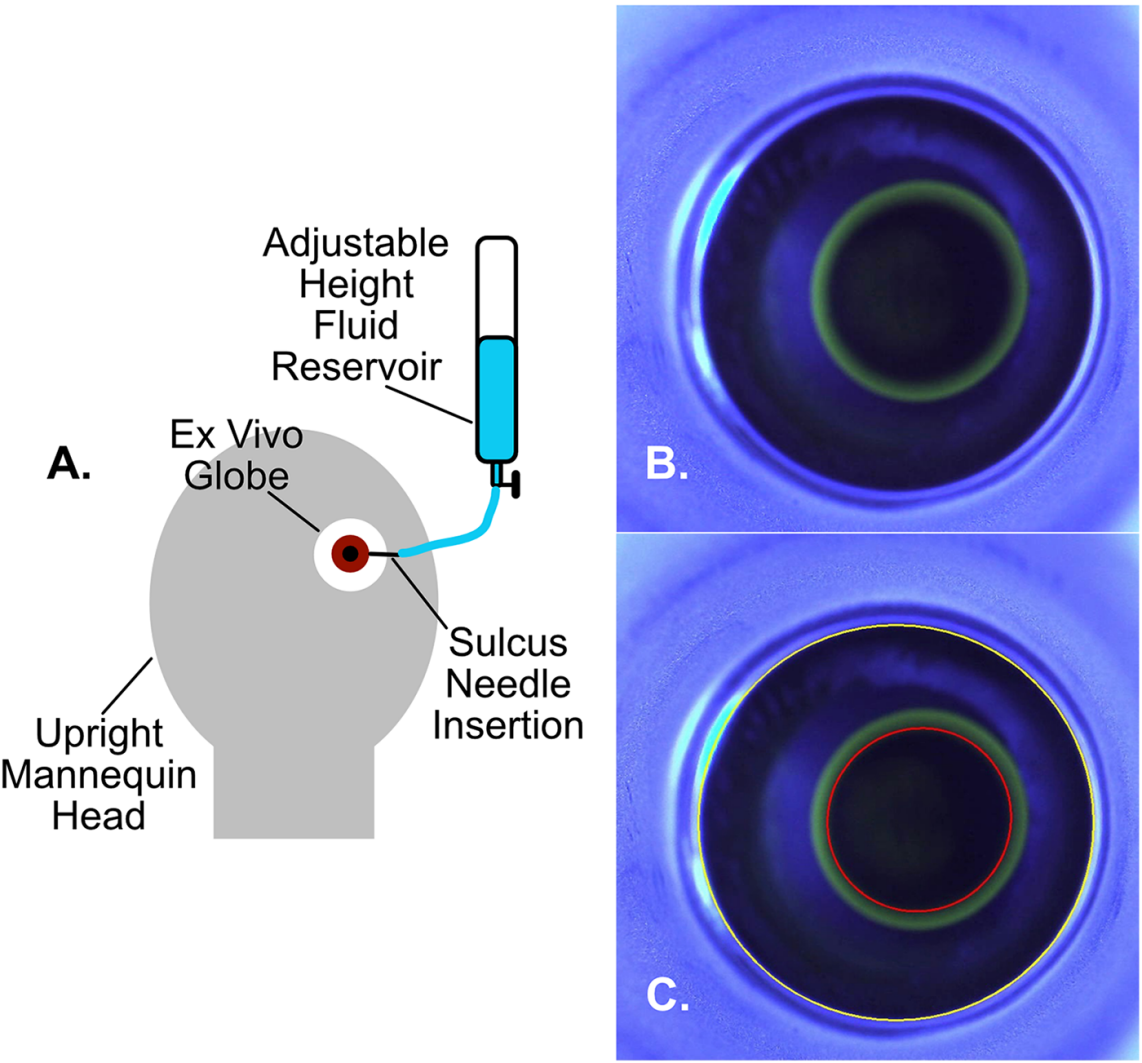
Experimental setup for IOP validation and image segmentation. (**A**) An ex vivo globe mounted in the upright position with a sulcus needle is perfused at various set heights to create IOPs ranging from 5 to 45 mm Hg. (**B**, **C**) An example frame from the applanation recording is converted to an IOP by identifying the reference tonometer tip (*yellow outer circle*) and applanation mire (*red inner ellipse*).

The human tissue experiments complied with the guidelines of the ARVO Best Practices for Using Human Eye Tissue in Research. Ten human cadaveric eyes (five pairs) were provided by the Miracles in Sight Eye Bank (Clemmons, NC) (Table). Eyes were collected and prepared within 24 hours of death. Whole eyes were mounted into a Styrofoam mannequin head to best approximate eyes in situ and clinic conditions while using the device (Fig. 2A). Pins were used to attach the ocular muscles to the Styrofoam without puncturing the globe itself. The superior quadrant was identified and placed in the 12 o'clock position within the Styrofoam.

**Table.** Donor Information

Age	Sex	Race	Time From Death to Preservation	Time From Death to Receipt	Cause of Death	Ocular History
77	Male	White	5 h, 22 min	7 h, 16 min	Respiratory failure	Glaucoma
83	Male	White	4 h, 40 min	11 h, 37 min	Heart disease	Unavailable
77	Male	White	4 h, 46 min	5 h, 57 min	Lung cancer	Unavailable
85	Male	White	7 h, 17 min	22 h, 3 min	GI bleed	Cataract surgery
83^a^	Female	White	7 h, 5 min	20 h, 27 min	Leukemia	Unavailable

a Excluded due to edematous corneas.

Following eye placement, the Styrofoam was positioned upright and a reservoir filled with warmed, degassed phosphate-buffered saline (PBS) with calcium and magnesium supplemented with 5.5-mM glucose (DBG) was placed at a height that would apply 5 mm Hg to the eye (6.8 cm H_2_O to establish reservoir height). Pressure tubing was attached to the reservoir, and a 25-gauge needle was attached to the other end of the tubing for cannulation. The tubing and needle were primed with warmed, degassed DBG from the reservoir. The sulcus was then cannulated at a pressure of 5 mm Hg, with a sulcus sclerae cannulation placing the needle just posterior and parallel to the iris. Eyes were allowed to rest at cannulation pressure (5 mm Hg) for 5 to 10 minutes prior to taking any measurements (equilibration).

After equilibration, topical fluorescein sodium 0.25%/benoxinate 0.4% (Flurox; Altaire Pharmaceuticals, Aquebogue, NY) was applied to the cornea, ensuring that the whole cornea was covered, and the applanator tip was placed in contact with the cornea until an applanation circle could be seen on the screen. The device was held to maintain a constant fixed force for 3 to 5 seconds in duration to ensure clear applanation images. Eyes were rinsed with DBG between each pressure step, and fresh fluorescein was applied before the next measurement was taken. The IOPs in mm Hg were converted to cmH_2_O, and the reservoir height was raised to assess IOP at 5, 10, 15, 20, 25, 30, 35, 40, and 45 mm Hg equivalents with at least 1 minute of equilibration time at each IOP prior to tonometry measurement.

### Image Analysis

The video processing algorithm calculates the median IOP of each video using a frame-by-frame analysis (Fig. 2B). Each frame is first cropped to remove dead space and converted from an red/green/blue (RGB) image to a grayscale image by isolating the blue color channel. The edge of the applanation prism is identified using Hough circle detection, and the frames are additionally cropped to this region (Fig. 2C). The applanation prism is periodically re-identified to maintain accurate cropping. Each frame then undergoes a contrast adjustment using Contrast Limited Adaptive Histogram Equalization (CLAHE) before creating a binary mask to identify potential applanation contours.^21^^,^^22^ Potential applanations are filtered by size, location, and eccentricity and are then approximated as ellipses using the starburst method.^23^ The starburst method works by starting at a diameter smaller than the expected ellipse radius, stepping out radially in 20 evenly spaced directions until the contour mask is reached, and fitting an ellipse to a consecutive subset of those points (Fig. 2C). Visual inspection of identified ellipses demonstrated that, if more than one ellipse was identified, the smallest was the one delineating the applanation mire, and the remaining large ellipses were due to imaging artifacts. Therefore, if more than one eligible ellipse is identified in a frame, the smallest is selected. The pressure can then be calculated using the applanation area, known prism area, and known constant force. These applanation frames are stored in a buffer where outlier frames are removed by mean-ratio bounds within an applanation, and outlier applanations are removed using *z*-scores until the standard deviation of applanations across a video is acceptable, ultimately producing a final median pressure of the entire video from all valid applanations.

Statistical analysis was performed using Prism 10 (GraphPad Software, Boston, MA). Statistical significance was defined as *P* < 0.05. Mean error and mean absolute error between set IOPs and device IOPs were calculated for the full 5- to 45-mm Hg range, as well as the more clinically relevant range of 10 to 30 mm Hg. Linear correlation was used to compare manometrically set IOP with prototype device–measured IOP.

## Results

The force output for the prototype device in both the vertical and horizontal position was similar and consistent with the force output for the GAT set at 2.5 gF. The mean ± SD was found to be 2.46 ± 0.12 gF for the prototype device used in the vertical position, 2.47 ± 0.12 gF for the prototype device used in the horizontal position, and 2.48 ± 0.11 gF for GAT set at 2.5 gF (Fig. 1C).

**Figure 3.**
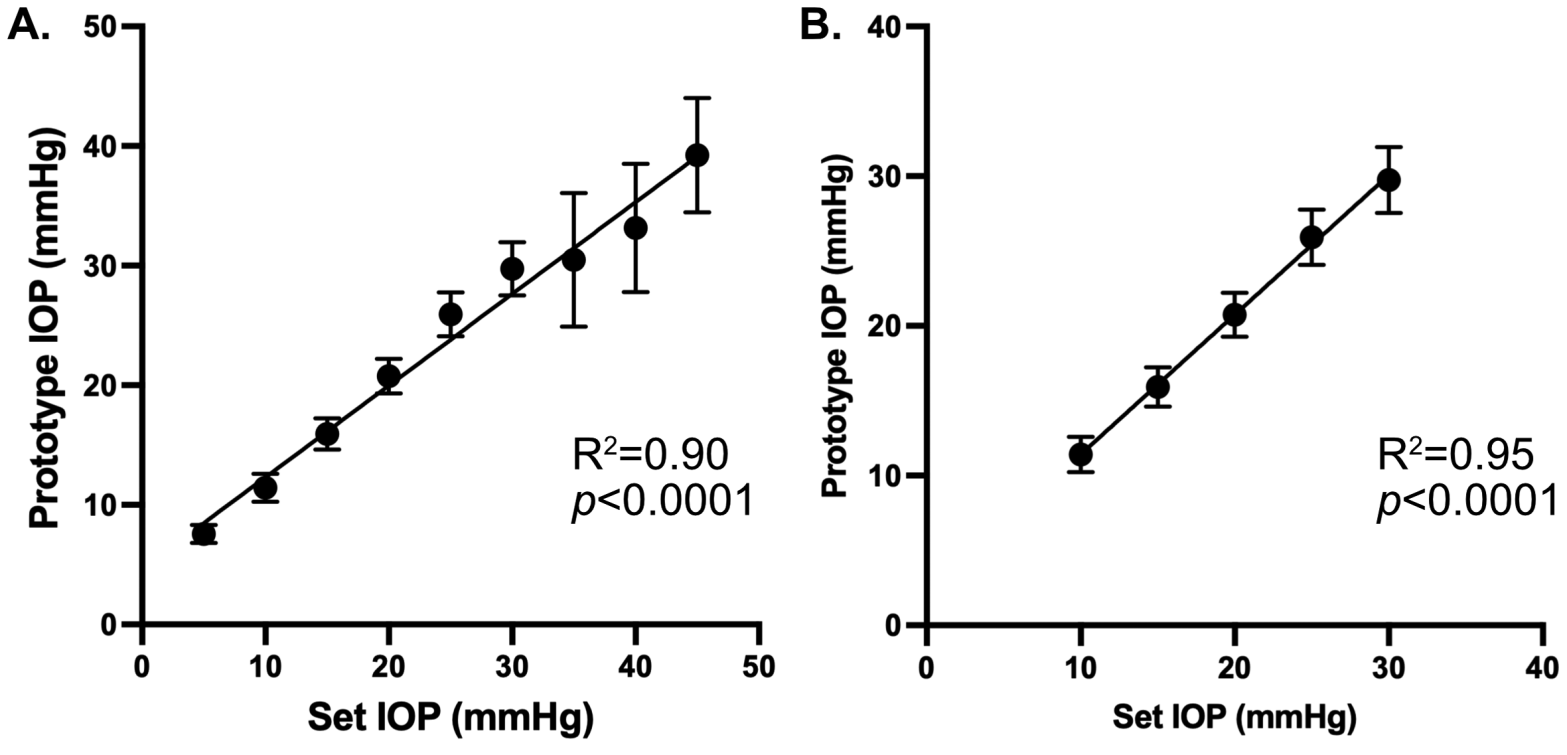
Comparison of the prototype device with the set IOPs. (**A**) A comparison is shown across the full range of set IOPs. The *solid line* represents the linear regression for the prototype, where IOP = 0.77 × (set IOP) + 4.6; slope = 0.77 (95% confidence interval [CI], 0.70–0.83); and *y*-intercept = 4.6 (95% CI, 2.9–6.4). (**B**) In the more clinically relevant range of 10 to 30 mm Hg, the *solid line* represents the linear regression for the prototype, where IOP = 0.93 × (set IOP) + 2.1; slope = 0.93 (95% CI, 0.86–1.0); and *y*-intercept = 2.1 (95% CI, 0.55–3.6).

The validation study included 10 ex vivo human globes from five donors. Two globes from a single donor were excluded due to significant corneal edema that precluded accurate applanation measurements because mires could not be clearly identified on a background of diffuse fluorescein staining. All 64 recordings on eight eyes (100%) were successfully processed and converted to IOP measurements. The mean ± SD difference and the mean absolute difference ± SD between the set IOP and the prototype IOP were –1.2 ± 4.5 mm Hg and 3.1 ± 3.5 mm Hg, respectively. Within the 10- to 30-mm Hg range, the mean difference and mean absolute difference between the set IOP and the prototype IOP were 0.7 ± 1.7 mm Hg and 1.4 ± 1.1 mm Hg, respectively. The prototype IOP and the set IOP showed a significant linear correlation across the full range of set IOPs (*R*^2^ = 0.90, *P* < 0.0001), which improved further when just examining the 10- to 30-mm Hg range of set IOPs (*R*^2^ = 0.95, *P* < 0.0001) (Fig. 3).

## Discussion

We developed a novel, handheld, automated applanation tonometer based on fixed-force applanation principles. The prototype device demonstrated comparable fixed-force output compared to a standard GAT tonometer set to the same applanating force. We then validated the prototype on a system where IOP was set manometrically and demonstrated a strong correlation between the prototype-measured IOP and the set IOP (*R*^2^ = 0.90). Within the 10- to 30-mm Hg range, which is most clinically significant, the correlation between the device and the set IOP was even stronger (*R*^2^ = 0.95).

In 1885, Maklakov developed the first fixed-force applanation tonometer, which involved resting an ink-coated weight under gravity on the supine cornea and then measuring the resultant area of ink transferred off the weight onto the cornea.^5^ Posner^24^ developed a subsequent iteration in 1964, which also utilized ink to transfer the applanated impression onto paper, which was then measured. Later, Halberg^25^ developed a tonometer where a clear weight was rested on the supine cornea and the applanated area was measured by direct visualization under a magnifying lens with a scaled ruler. However, accurate measurement of the applanated area under fixed-force techniques proved to be challenging, with a significant learning curve, and these techniques were largely abandoned with the development and popularity of GAT.

Improvements in software and image processing have made it possible to accurately and easily identify and measure applanation areas. Mariakakis et al.^26^ described a smartphone-based system that applied fixed-force principles and used algorithmic estimations of applanation area to calculate IOP. Briefly, a clear 5-g weight was applied to an eye coated in fluorescein, and, under blue light, the smartphone camera was used to record the resulting applanation image, which was then converted to an IOP. This system was tested on ex vivo porcine eyes, with a strong correlation to the manometrically set pressure (*r* = 0.89). A subsequent smartphone prototype was developed and tested in a clinical study comparing its measurements to other clinical tonometers. Using machine learning to better calculate the area of the tonometer circle produced by applanation, this prototype demonstrated a moderate correlation to GAT (*r* = 0.82).^27^

Our current prototype improves upon the prior work by allowing measurements in the upright position with better standardization of the blue LED illumination to create a more consistent applanation appearance. Compared to the prior smartphone prototype where fewer than 57% of recordings were successfully processed, in the ex vivo eyes where the corneas had not deteriorated (*n* = 2), we were able to successfully process 100% of the recorded videos in this study (*n* = 8 eyes, 64 total recordings). Across the full range of set IOPs (5–45 mm Hg), the mean deviation of the prototype was –1.2 ± 4.5 mm Hg, with a correlation coefficient of *r* = 0.95. This compares favorably to a study by Ertel et al.^28^ comparing manometrically set IOPs in ex vivo human eyes to various commercially available portable tonometers, including the Perkins (Haag-Streit, Hertfordshire, UK), iCare 200 (iCare Finland Oy, Vantaa, Finland), and Tono-Pen (Reichert Technologies, Depew, NY). In this study, the Perkins tonometer had the most accurate performance, with a mean difference of –1.0 ± 5.0 mm Hg compared to the manometric IOP, whereas the mean difference for the iCare 200 was 5.3 ± 2.2 mm Hg and for the Tono-Pen it was 4.6 ± 4.0 mm Hg. Similarly, the mean absolute difference for the prototype (3.1 ± 3.5 mm Hg) also compared favorably to the Perkins (4.0 ± 3.1 mm Hg), iCare 200 (5.3 ± 2.2 mm Hg), and the Tono-Pen (5.4 ± 2.8 mm Hg).

The normal physiologic IOP has historically been defined as 21 mm Hg or less, representing the 2-SD threshold above the population average.^29^ Even for the vast majority of glaucoma patients, IOP can be expected to fall within the 10- to 30-mm Hg range. For example, a large cross-sectional study showed that the mean IOP in glaucoma patients was 20.2 mm Hg with a SD of 5.4 mm Hg.^30^ Performance of tonometers in the 10- to 30-mm Hg range is therefore of critical importance in clinical care.

Within the 10- to 30-mm Hg range, the prototype measurements are highly correlated (*r* = 0.97) with a reduced mean difference of 0.7 ± 1.7 mm Hg. This was less than the reported mean differences for the Perkins (1.1 ± 2.7 mm Hg), iCare 200 (5.8 ± 2.2 mm Hg), and Tono-Pen (4.2 ± 2.1 mm Hg) tonometers for the same range.^28^ Similarly, the mean absolute difference in the 10- to 30-mm Hg range for the prototype (1.4 ± 1.1 mm Hg) was also less than the reported absolute differences for the Perkins (2.2 ± 1.8 mm Hg), iCare 200 (5.8 ± 2.2 mm Hg), and Tono-Pen (4.2 ± 2.1 mm Hg) tonometers. Importantly, Ertel et al.^28^ reported that, in their study, the Perkins tonometer had the least mean difference compared to manometry but had the greatest variability compared to the other handheld tonometers. Within 10 to 30 mm Hg, our prototype measurements had both the least mean difference and least variability compared to the reported literature. Asrani et al.^29^ argued that tonometric precision is more important than accuracy in clinical care of glaucoma patients. These data suggest that this prototype would be an improvement on *both* precision and accuracy compared to existing handheld tonometers.

Like our tonometer, outside the 10- to 30-mm Hg range most handheld tonometers become less accurate. The Tono-Pen has been observed to overestimate IOP in the lower ranges and underestimate at the higher ranges.^31^^,^^32^ Similarly, the iCare tonometer has been observed to underestimate IOP compared to GAT when GAT measurements are above 23 mm Hg.^33^ Our data suggest that our prototype tends to overestimate IOP measurements below 10 mm Hg and underestimate IOP above 30 mm Hg. This shift toward improved accuracy is also reflected in the respective linear equations, where the slope improves from 0.77 for the full range of set IOPs to 0.93 for the 10- to 30-mm Hg range (where a slope of 1.0 would be perfect accuracy). Fixed-force applanation has long been observed to be affected by changes in the ocular rigidity, which is dependent on the IOP. The Halberg and Posner fixed-force tonometers included additional 7.5-g and 10-g weights to be used on eyes with higher IOPs. Interestingly, prior publications on these tonometers have reported ideal IOP performance when the applanation diameters were 3.8 to 5.6 mm (Halberg) or 4.0 to 5.5 mm (Posner), which correspond to 13 to 36 mm Hg and 13 to 31 mm Hg, respectively.^24^^,^^25^ Above that range, the higher weights are recommended. Our prototype has a similar ideal range for measurement, and future iterations may include the option to increase the applied force to improve accuracy at higher IOPs. Although the current device is designed to produce a constant force of 2.5 gF, theoretically a device could be designed with a method for adjusting the spring preload and counterweight location and mass to create other fixed forces, but this has not yet been developed or tested.

The Perkins tonometer is also a portable, handheld applanation-based tonometer but differs from our prototype. Mechanically, the Perkins tonometer varies the applied force, and the operator must manually adjust the force dial until the semicircular mires within the applanation prism are optically aligned.^12^ Conversely, our device applies a fixed force and measures the applanation area through the prism using a camera. The Perkins tonometer uses a gravity-following pendulum counterweight to keep the prism axis pointed at the cornea regardless of device orientation,^12^ whereas our device uses a tuned counterweight to make the gravity moment about the pivot point match in the vertical and horizontal device orientations. Like the Perkins, our device can be used in upright and supine positions but the automated conversion of applanation area to IOP reduces the operator-introduced reading errors and improves accuracy and repeatability.

Additional testing and assessment of long-term device stability will be necessary to determine the optimal frequency of calibration for the device. Damage to the device during shipping, daily use, and changes in temperature and humidity could cause the constant force applied by the device to drift over time. The mechanical behavior of the spring may change over time due to spring creep. In this device, the spring experiences less than 10 gF, making it unlikely to wear out quickly from cyclical stressing. Music wire springs may relax by 1% to 2% at room temperature over their lifespan. Of note, a 1% increase in spring length would result in only about a 0.04-gF decrease in force applied by the device, which is unlikely to significantly alter measurement accuracy. However, future studies are needed to quantify the device performance over a simulated device lifetime to understand how often calibration may be needed.

There are also several limitations to this study. The sample size of eight ex vivo eyes was relatively small but consistent with other published studies using ex vivo cadaveric globes.^28^^,^^34^^,^^35^ Also, although ex vivo eyes have the experimental advantage of precise pressure control, they may possess different properties than in vivo eyes such as postmortem corneal edema, corneal elasticity, tear-film surface tension, and corneal hysteresis. Each of these factors has a material impact on IOP measurements.^36^^,^^37^ Encouragingly, McCafferty et al.^35^ reported that assessment of tonometric performance using perfused fresh human cadaver eyes compared well to intra-surgical live human eyes, suggesting that our findings should translate into clinical practice reasonably well. Only male eyes were available to complete this study (one pair of female eyes was excluded due to corneal edema), which could be a potential source of bias. No pachymetry or other corneal data were collected in our study. Although the absence of corneal biomechanical data limits our generalizability to other corneas, it also suggests that our reported accuracy is conservative; mathematically adjusting these uncorrected IOP values could theoretically improve our results.^38^ The Imbert–Fick principle assumes a thin-walled sphere, and the biomechanical properties or thickness of the cornea itself may contribute to the residual projected prototype IOP, even when the set IOP is 0 mm Hg (as noted by the *y*-intercept of 2.1 mm Hg in the linear equation). Finally, the measurements obtained in this study likely benefited from the lack of patient movement and better operator stability in a laboratory setting. Future studies should address these limitations by incorporating larger sample sizes of in vivo eyes of both sexes and collecting pachymetry and other corneal metrics. As GAT has proven its value to the ophthalmic community and its patients for over 75 years, future studies should evaluate the usability, affordability, and patient acceptance of this tonometer to better determine if this is a superior alternative or helpful adjunct.

## Conclusions

Our novel, handheld, automated, fixed-force applanation tonometer is an accurate tool for measuring IOP. This device addresses several limitations of traditional GAT, including the subjective alignment of mires and the substantial learning curve. The results in ex vivo human globes are promising, particularly at physiologic IOPs. Future studies should focus on clinical validation in living participants and evaluate the performance of the device across corneas with varying biomechanical properties.

## Supplementary Material

Supplement 1

Supplement 2
