# Supplementary material for: Validation of a New Handheld Automated Fixed-Force Applanation Tonometer
Source: Transl Vis Sci Technol. 2026 Jul 14;15(7):21. doi: 10.1167/tvst.15.7.21 (PMC13387270; doi:10.1167/tvst.15.7.21)
Supplement: Supplement 1 [file tvst-15-7-21_s001.docx]

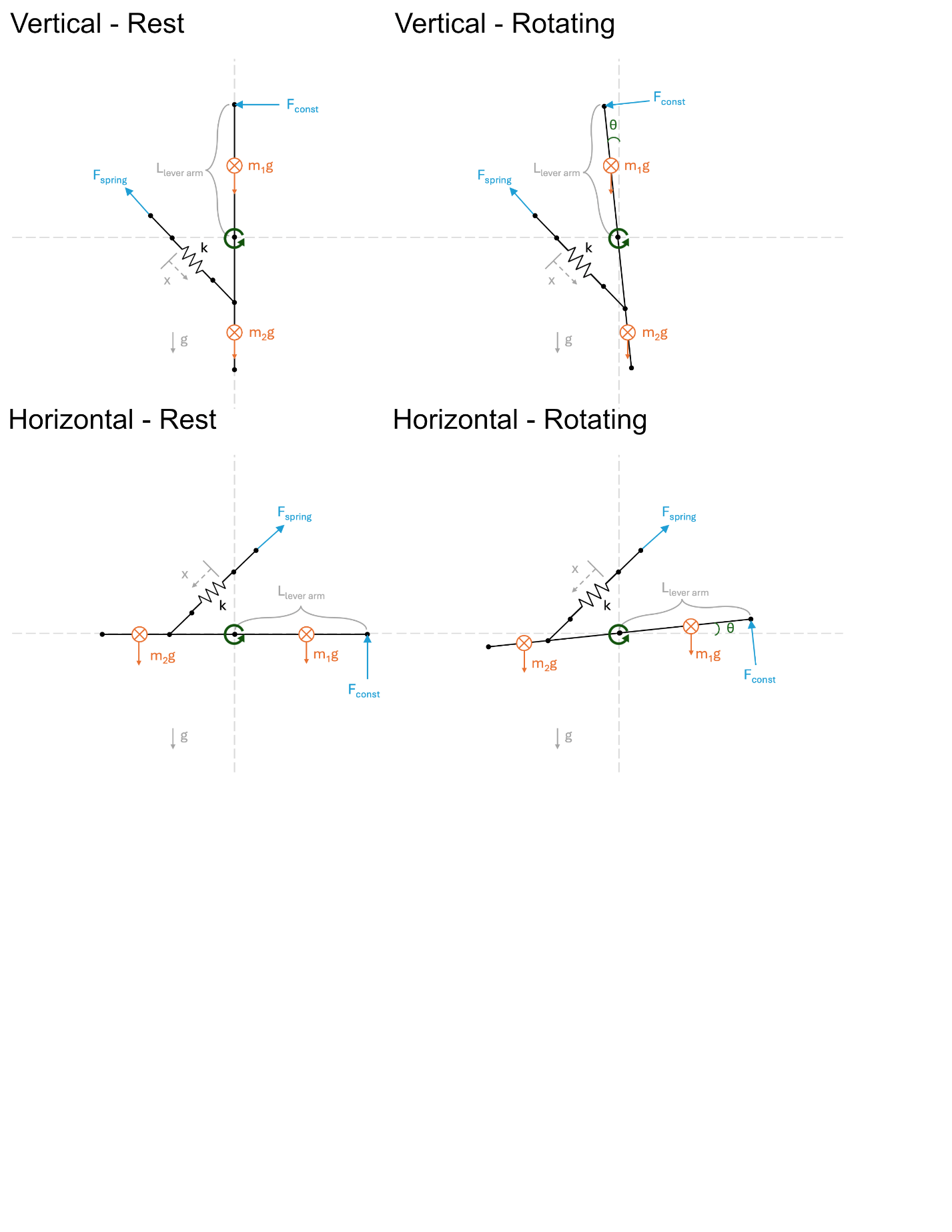
Supplemental Figure. Free body diagrams for the prototype device in the vertical and horizontal position while resting and rotating.
